# Angiotensin-Receptor Blocker, Angiotensin-Converting Enzyme Inhibitor, and Risks of Atrial Fibrillation: A Nationwide Cohort Study

**DOI:** 10.1097/MD.0000000000003721

**Published:** 2016-05-20

**Authors:** Yu-Cheng Hsieh, Chen-Ying Hung, Cheng-Hung Li, Ying-Chieh Liao, Jin-Long Huang, Ching-Heng Lin, Tsu-Juey Wu

**Affiliations:** From the Department of Internal Medicine (Y-CH), Chiayi Branch, Taichung Veterans General Hospital, Chiayi; Cardiovascular Center, Taichung Veterans General Hospital and Department of Internal Medicine (Y-CH, C-YH, C-HL, Y-CL, J-LH, T-JW), Faculty of Medicine, Institute of Clinical Medicine, Cardiovascular Research Center, National Yang-Ming University School of Medicine, Taipei; Department of Financial and Computational Mathematics (Y-CH), Providence University, Taichung; Department of Internal Medicine (C-YH), Hsinchu Branch, Taipei Veterans General Hospital, Hsinchu; Department of Nutrition (C-YH), Hungkuang University; School of Medicine (J-LH), Chung Shan Medical University; and Department of Medical Research (C-HL), Taichung Veterans General Hospital, Taichung, Taiwan.

## Abstract

Both angiotensin-receptor blockers (ARB) and angiotensin-converting enzyme inhibitors (ACEI) have protective effects against atrial fibrillation (AF). The differences between ARB and ACEI in their effects on the primary prevention of AF remain unclear. This study compared ARB and ACEI in combined antihypertensive medications for reducing the risk of AF in patients with hypertension, and determined which was better for AF prevention in a nationwide cohort study.

Patients aged ≥55 years and with a history of hypertension were identified from Taiwan National Health Insurance Research Database. Medical records of 25,075 patients were obtained, and included 6205 who used ARB, 8034 who used ACEI, and 10,836 nonusers (no ARB or ACEI) in their antihypertensive regimen. Cox regression models were applied to estimate the hazard ratio (HR) for new-onset AF.

During an average of 7.7 years’ follow-up, 1619 patients developed new-onset AF. Both ARB (adjusted HR: 0.51, 95% CI 0.44–0.58, *P* < 0.001) and ACEI (adjusted HR: 0.53, 95% CI 0.47–0.59, *P* < 0.001) reduced the risk of AF compared to nonusers. Subgroup analysis showed that ARB and ACEI were equally effective in preventing new-onset AF regardless of age, gender, the presence of heart failure, diabetes, and vascular disease, except for those with prior stroke or transient ischemic attack (TIA). ARB prevents new-onset AF better than ACEI in patients with a history of stroke or TIA (log-rank *P* = 0.012).

Both ARB and ACEI reduce new-onset AF in patients with hypertension. ARB prevents AF better than ACEI in patients with a history of prior stroke or TIA.

## INTRODUCTION

Atrial fibrillation (AF) is the most common arrhythmia and is associated with high mortality and morbidity.^1^ Hypertension is the most common risk factor and is associated with a 40% to 50% increased risk of developing new-onset AF.^2^ AF has become more prevalent with the increase in the elderly population in recent years.^1^ Therefore, effective prevention for new-onset AF in hypertensive patients is a major issue in disease management.^1^ Angiotensin II, oxidative stress, and proinflammatory mediators are important factors which induce atrial remodeling and ectopic activities in pulmonary veins, leading to AF occurrence.^3^ Increasing evidence suggests that upstream therapies, such as angiotensin-converting enzyme inhibitor (ACEI), angiotensin-receptor blocker (ARB), statin, and aldosterone antagonist can be used for AF prevention.^4^ Among the upstream therapies, both ACEI and ARB are recommended for AF prevention in clinical guidelines because they might modify atrial substrate, prevent inflammation, and thus reduce the risk of AF.^3–5^

Although ACEI and ARB inhibit the renin–angiotensin system by targeting different sites in the pathway, clinical studies have shown that both drugs effectively lower blood pressure (BP) and reduce cardiovascular events.^6–8^ Clinicians therefore regard ACEI and ARB as effectively equivalent in their ability to provide cardiovascular protection.^9^ However, the comparative effectiveness of ACEI versus ARB in preventing new-onset AF in hypertensive patients is rarely reported.^3^ We previously found that ACEI/ARB and mixed users have a lower risk of AF than nonusers, although we did not directly compare the effects of ACEI versus ARB on AF risk.^10^ A previous cohort study showed that ARB monotherapy was significantly better than ACEI monotherapy on AF prevention in hypertensive patients.^11^ Whether ARB is better than ACEI for primary AF prevention in hypertensive patients receiving multiple antihypertensive medications remains controversial. The purpose of the present study was to evaluate: if ARB or ACEI use as one of the combined antihypertensive medications reduced the risk of AF compared with non-ACEI/ARB users; and whether ARB is better than ACEI for primary prevention of AF in patients with hypertension in a nationwide cohort. We also evaluated if any cardiovascular comorbidity could predict the comparative effectiveness of ARB and ACEI for primary AF prevention.

## MATERIALS AND METHODS

### Research Database

The National Health Insurance program in Taiwan was implemented in 1995, and currently nearly 99% of the Taiwanese population is enrolled in this program. The National Health Research Institutes (NHRI) established the National Health Insurance Research Database. In this study, we used a systemic sampling of patients’ data from 2000 to 2011 with a total of 1,000,000 subjects in a dataset released by the NHRI. The NHRI has confirmed that this random sample is representative of the general Taiwanese population, and thus there were no statistical differences in age and gender between the general population and the study sample. The database includes outpatient visits, hospital admissions, prescriptions, and disease records. The NHRI safeguards the privacy of the patients in the database and provides data to researchers only after ethical approval has been verified. Therefore, patients’ data are provided by the NHRI in an anonymous format, such that specific individuals cannot be identified.^10^ This study was approved by the Institutional Review Board of Taichung Veterans General Hospital.

### Study Population

Patients aged ≥55 years and with a diagnosis of hypertension were identified according to the International Classification of Diseases, Ninth Revision, Clinical Modification (ICD-9-CM) code 401-405 in 2003. To avoid misclassification and to validate the diagnosis, only patients who had a diagnosis of hypertension and had used at least 1 antihypertensive drug were selected for analysis (n = 57,146). As the primary focus of the investigation was new-onset AF, patients (n = 7037) were excluded if they had a history of cardiac arrhythmias in 2003. Those who (n = 25,034) had ever used both ACEI and ARB, either sequentially or concomitantly, were also excluded. Thus, there were 3 groups of patients in the final analysis: those who used ACEI alone (n = 8034), ARB alone (n = 6205), and nonusers (n = 10,836).

### Definitions of Drug Use

Drug usage information, including prescribed drug types, dosage, date of prescription, and total number of pills dispensed, was obtained from an ambulatory and inpatient claims database. The ARB and ACEI use records, prescription dates, and the number of pills per prescription were collected for analysis. Patients who used ARB or ACEI for more than 28 days were defined as ARB or ACEI users, while those with mixed use of ARB or ACEI were excluded from this study. We divided the patients into 3 groups, those who used ARB alone, ACEI alone, and nonusers, according to their ARB or ACEI usage between January 1, 2003, and the event date (if AF occurred), or December 31, 2011 (if no AF occurred).

### Study Endpoint

The endpoint of this study was new-onset AF (ICD-9-CM code 427.31) during the 8-year follow-up period (2004–2011). The occurrences of AF were identified using the claims data. In the analysis, we included patients with diagnosis of AF in at least 3 consecutive outpatient visits (to exclude those with a tentative AF diagnosis, who were just receiving an AF examination or retrieving a report), or at least 1 hospitalization in which AF was one of the final diagnoses at discharge. Patients were followed from January 1, 2004 to the study endpoint, that is, occurrence of AF, or study termination (December 31, 2011).

### Covariate Ascertainment

Demographic data including age and sex were recorded. We identified cardiovascular comorbidities as potential confounders by using ICD-9-CM codes between January 1, 2003 and December 31, 2003. Inpatient and outpatient files were used to ascertain the comorbidities including heart failure, diabetes mellitus, stroke or transient ischemic attack (TIA), vascular disease, thyroid disease, valvular heart disease, chronic obstructive pulmonary disease, and renal disease.

### Statistical Analysis

The data are presented as mean ± standard deviations for continuous variables, and proportions for categorical variables. Analysis of variance and Chi-square tests were used for comparing differences in continuous and categorical variables. The AF-free survival curves were plotted by the Kaplan–Meier method, and the statistical significance was examined by log-rank test. Multivariable Cox proportional hazard regression, which was used to estimate the association between ACEI/ARB use and the occurrence of AF, was expressed by hazard ratio (HR) and 95% confidence interval (CI). All statistical analyses were carried out by SAS software version 9.2 (SAS Institute, Inc., Cary, NC). A *P* value of <0.05 was considered statistically significant.

## RESULTS

### Baseline Characteristics

A total of 25,075 hypertensive patients were enrolled in this study. Table 1 shows the baseline characteristics of ARB users, ACEI users, and nonusers. ARB users (68.4 ± 8.0 years) were younger than ACEI users (69.8 ± 8.7 years) and nonusers (70.2 ± 8.9 years) (*P* < 0.001), and the percentage of females was higher in ARB users (54.9%) than that in ACEI users (47.7%) and nonusers (51.3%) (*P* < 0.001). Diabetes mellitus was the most prevalent medical disease in ARB (18.2%) and ACEI (21.8%) users, while stroke/TIA (14.7%) was the predominant associated disease in nonusers.

**TABLE 1:**
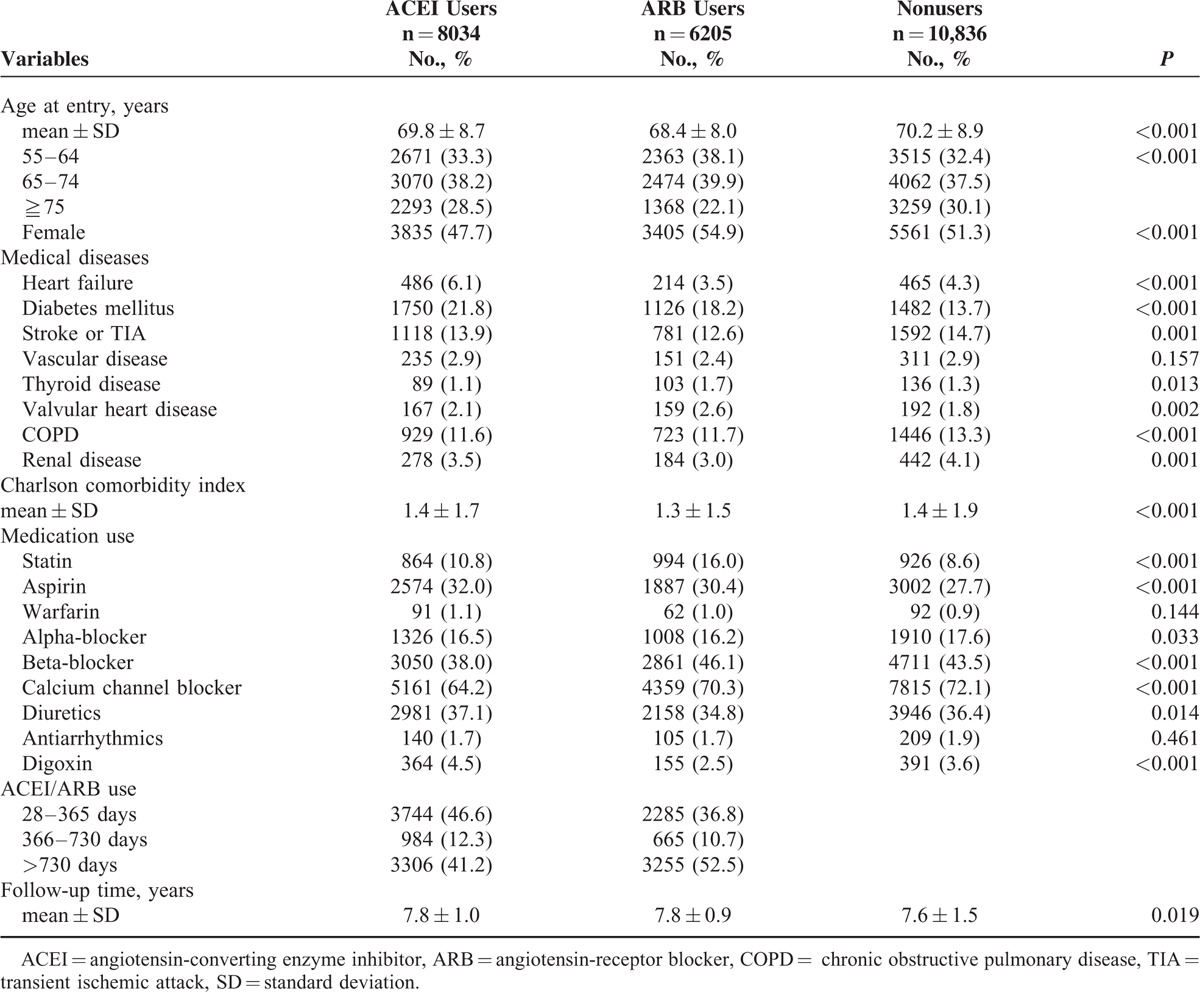
Baseline Characteristics of the Patients

Compared to ARB users, ACEI users had a higher prevalence of heart failure, diabetes mellitus, stroke/TIA, vascular disease, and renal disease, but had a lower prevalence of thyroid, valvular heart diseases, and chronic obstructive pulmonary disease. Calcium channel blocker was the most frequently prescribed antihypertensive medication, followed by beta-blocker, which was the second most commonly used antihypertensive drug in these 3 groups. The percentage of statin users was higher in ARB (16.0%) and ACEI (10.8%) users than that in nonusers (8.6%) (*P* < 0.001). The percentages of antiarrhythmic drug users (*P* = 0.461) and warfarin users (*P* = 0.144) were evenly distributed among these 3 groups. The follow-up duration was not significantly different between ARB (7.8 ± 0.9 years) and ACEI (7.8 ± 1.0 years) users.

### Effects of ARB and ACEI on AF Prevention

During an average of 7.7 years’ follow-up, a total of 1619 new-onset AF occurred. The overall incidence was 8.4/1000 person-years. Table 2 shows the HRs for new-onset AF in the cohorts. Compared to nonusers (11.7/1000 person-years), the incidence of new-onset AF was lower in ARB (5.6/1000 person-years, adjusted HR: 0.51, 95% CI 0.44–0.58, *P* < 0.001) and ACEI users (6.2/1000 person-years, adjusted HR: 0.53, 95% CI 0.47–0.59, *P* < 0.001) before and after adjustments for variables between the cohorts. Table 2 also shows the duration of ARB/ACEI use and the risk of AF. As the duration of ARB/ACEI use increased, the incidence of AF progressively decreased. For ACEI users, the incidence rates of AF were 6.9/1000, 5.8/1000, and 5.4/1000 person-years for the treatment durations of 28–365, 366–730, and >730 days, respectively. Similarly, the incidence rates of AF were 7.8/1000, 6.4/1000, and 3.9/1000 person-years in patients who used ARB for 28–365, 366–730, and >730 days, respectively.

**TABLE 2:**
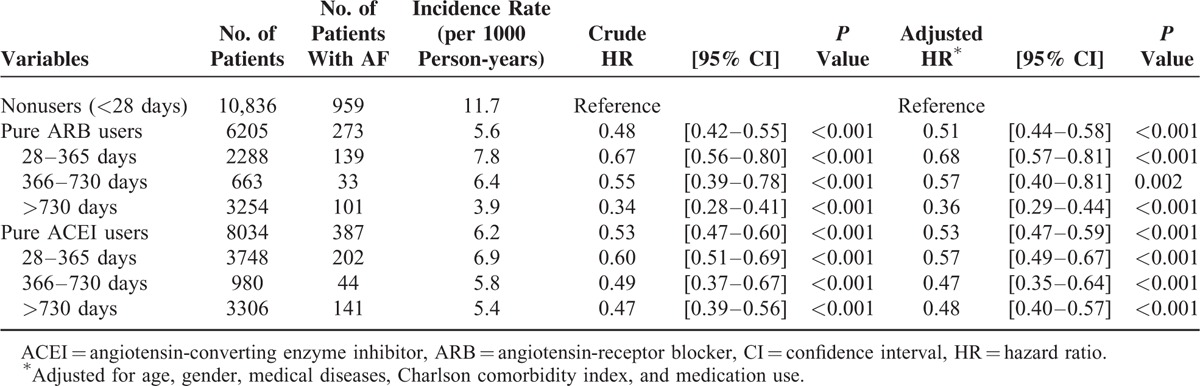
Dose Relation Analysis for New-Onset Atrial Fibrillation

### Comorbidities and Treatment Outcome

**FIGURE 1:**
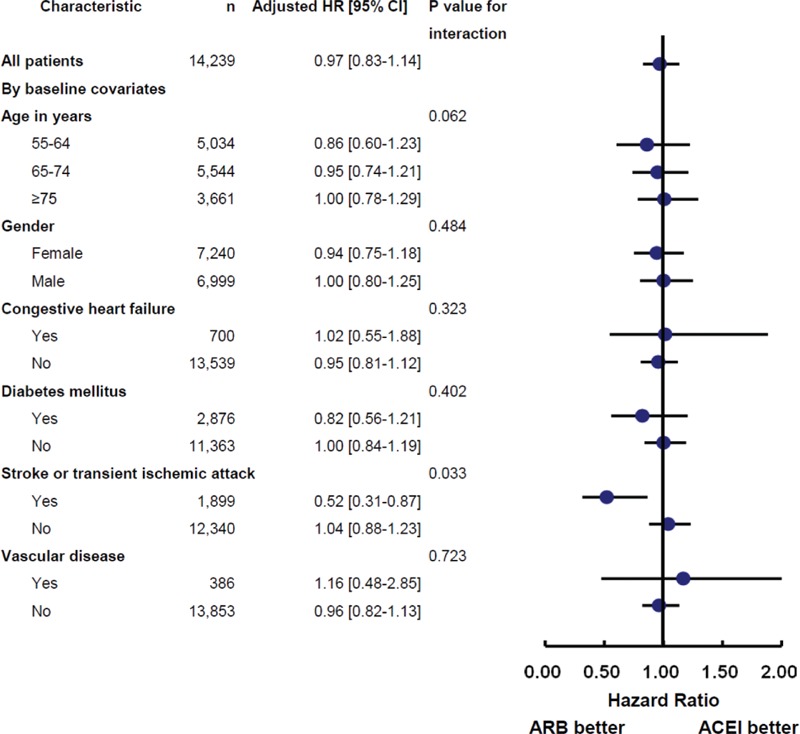
Subgroup analysis comparing new-onset atrial fibrillation in patients using ARB or ACEI. ACEI = angiotensin-converting enzyme inhibitor, ARB = angiotensin-receptor blocker.

Figure 1 shows subgroup analysis comparing ARB versus ACEI use in preventing new-onset AF by Cox proportional hazards analysis. There were no significant differences in AF hazard risk ratios between ARB and ACEI users with regard to age, gender, congestive heart failure, diabetes, and vascular disease (*P* = ns for interaction in these subgroups). However, in patients with prior stroke or TIA, ARB users (adjusted HR: 0.52, 95% CI 0.31–0.87, *P* = 0.012) had a lower risk of AF than that (adjusted HR: 1.04, 95% CI 0.88–1.23, *P* = 0.628) of ACEI users after adjustments for age, gender, medical diseases, Charlson comorbidity index, and medication used (*P* value interaction 0.033).

**FIGURE 2:**
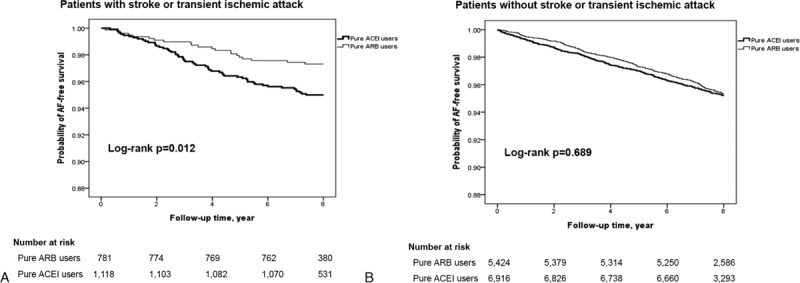
Atrial fibrillation-free survival rate in patients with (A) or without (B) prior stroke or transient ischemic attack.

Figure 2 shows the Kaplan–Meier survival plot comparing the AF-free survival rate between ARB and ACEI users in the presence (Figure 2A) or absence (Figure 2B) of prior stroke/TIA. In hypertensive patients with a history of stroke or TIA, ARB users had a lower incidence of AF than that of ACEI users (Figure 2A, log-rank *P* = 0.012). The survival curves began to separate early (at ∼2 years) and continued to separate throughout the entire course of this study. However, in hypertensive patients without a history of stroke or TIA, the incidence of AF was similar between ARB and ACEI users (Figure 2B, log-rank *P* = 0.689).

## DISCUSSION

There were 2 main findings in this study: both ARB and ACEI prevent new-onset AF in hypertensive patients receiving ARB/ACEI as one of the combined antihypertensive medications; ARB prevents new-onset AF better than ACEI in patients with prior stroke or TIA.

### ARB and ACEI Use in AF Prevention

Hypertension is the most prevalent and potentially modifiable risk factor for the occurrence of AF.^12^ Lowering BP per se by antihypertensive medication may reduce the risk of AF.^3,13^ Among all classes of antihypertensive medication, ACEI and ARB are preferred for AF prevention owing to their favorable effect on atrial remodeling, in addition to their BP-lowering effect.^4^ Clinical hypertension trials investigating the effects of ACEI and ARB on the risk of AF have generated conflicting results.^14–17^ However, meta-analysis data suggested that ACEI and ARB might prevent new-onset AF only in patients with left ventricular dysfunction and hypertrophy.^18,19^ Therefore, nationwide cohort studies with a large number of patients, a long observation period, and real-world prescription patterns might provide important information regarding whether ACEI and ARB can effectively prevent AF in hypertensive patients. Two nationwide cohort studies comparing ACEI or ARB monotherapy (excluding mixed ACEI/ARB users) to other classes of antihypertensive treatment consistently showed that ACEI and ARB are each associated with reduced risk of AF.^11,20^ In these cohort studies, patients were limited to using a single class of antihypertensive medications, and those with risk factors for developing AF, such as heart failure, diabetes mellitus, coronary heart disease, and thyroid disease, were excluded.^11,20^ The enrolment criteria indicated that the patients in the studies had mild hypertension and few cardiovascular comorbidities. In the current study, we enrolled patients with risk factors for AF, and allowed either ACEI or ARB as one of the multiple antihypertensive combinations for moderate and severe hypertensive patients. Therefore, the incidence of AF was higher in our study (5.6/1000 and 6.2/1000 person-years, for ARB and ACEI users, respectively) than that in a Danish nationwide study (1.5/1000 and 1.2/1000 person-years, for ARB and ACEI users, respectively).^11^ Despite the differences in study design and patients’ characteristics, we also found that both ACEI (adjusted HR: 0.53, *P* < 0.001) and ARB (adjusted HR: 0.51, *P* < 0.001) reduced the risk of new-onset AF by ∼50% in hypertensive patients. In this study, antiarrhythmic medications were minimally and evenly distributed among the 3 patient groups, suggesting that antiarrhythmic medication might not be the cause of reduced AF risk in ACEI or ARB users. We also found the longer the duration of ACEI or ARB use, the lower the risk was for the occurrence of AF. Previous nationwide studies conducted in Denmark and England as well as the present study demonstrate that using ACEI/ARB either as a monotherapy or as a combined with another antihypertensive medication can effectively reduce the risk of AF in hypertensive patients with or without risk factors for AF.

### ARB Versus ACEI in AF Prevention

Although both ARB and ACEI block the renin–angiotensin system and effectively lower BP in patients with hypertension, they produce different pathophysiological changes because they target different sites in the pathway.^6–8^ For example, ACEI may not completely inhibit angiotensin II production because of some unaffected converting enzymes, while ARB can directly block the angiotensin II type 1 receptor (AT1R).^8^ Furthermore, ACEI use is associated with adverse effects, including cough and angioedema not shared by ARB, while ARB might induce compensatory enhancement of angiotensin II type 2 receptor (AT2R) activities.^21^ Despite these mechanistic differences, a large-scale meta-analysis comparing ARB versus ACEI for BP reduction and cardiovascular outcomes proved them to be equally effective.^6,7^ However, new-onset AF is rarely considered as an outcome in these comparisons of ARB and ACEI. The only prospective randomized ONTARGET trial showed that ARB (telmisartan) did not reduce new-onset AF compared to ACEI (ramipril) in patients with high cardiovascular risks.^22^ Recently, Marott et al^11^ in a Danish cohort showed that ARB monotherapy showed better prevention of new-onset AF than ACEI monotherapy (HR: 0.68, CI 0.49–0.96, *P* = 0.04) in hypertensive patients without risk factors for AF. In contrast, we compared ARB and ACEI for AF prevention in hypertensive patients with multiple risk factors for AF, including congestive heart failure, diabetes, thyroid disease, valvular heart disease, and vascular diseases. We found that ARB prevented new-onset AF better than ACEI, specifically in hypertensive patients with a history of prior stroke or TIA, which was similar to the result of the Danish study. Our study included patients receiving combined antihypertensive medication including ACEI/ARB, which is a more realistic practice than using ARB or ACEI as a single antihypertensive drug, as reported in other studies.^11^ To the best of our knowledge, this is the 1st study to compare the effect of ARB versus ACEI use on AF prevention in patients with multiple risk factors for AF. Further studies are warranted to verify whether ARB use is better than ACEI in preventing AF in other subgroups of patients.

Several mechanisms might explain why ARB prevents AF better than ACEI in hypertensive patients. First, selective inhibition of AT1R by ARB may lead to compensatory increases in angiotensin II concentration, allowing free angiotensin II to bind to AT2R.^8,23^ Stimulation of AT2R has been reported to enhance nitric oxide secretion, which is associated with decreased AF in patients with cerebral infarction.^24–26^ Second, experimental studies showed that some ARBs, that is, candesartan and irbesartan, 2 commonly used ARBs in Taiwan, possess direct antiarrhythmic properties.^13,27^ Candesartan and irbesartan use in the ARB group might have contributed to the reduced AF risk in this study. Finally, in patients with prior stroke or TIA, sympathetic activation might elevate the BP either by increased catecholamine release or over-activation of the renin–angiotensin–aldosterone system.^28^ Two randomized trials showed that ARB use was associated with lower BP than that achieved by ACEI (0.9 mm Hg lower in ONTARGET trial, 4.0 mm Hg lower in DETAIL trial).^22,29^ In our patients with prior stroke, the use of ARB might have lowered the BP by more than that which was achieved by ACEI, as observed in previous clinical trials, and thus contributed to the lower AF risk.

### Study Strength

Few studies have investigated the differences in effects between ARB and ACEI on primary prevention of AF because both drugs are equally effective in reducing BP and cardiovascular outcomes.^6,7^ A Danish cohort study found that ARB monotherapy prevents new-onset AF better than ACEI monotherapy in hypertensive patients without risk factors for AF.^11^ In contrast to the aforementioned study, our study provides robust evidence that in hypertensive patients with risk factors for AF and who received ARB or ACEI as one of the medications in antihypertensive combination therapy, use of ARB alone prevented new-onset AF better than use of ACEI alone, especially in patients with prior stroke. We also showed that the longer the duration of ARB or ACEI use, the lower the risk was for the occurrence of AF.

## LIMITATIONS

Some limitations exist in this study. First, this was a retrospective cohort study. We could not be certain whether patients complied well with their prescribed medications. Second, the actual BP achieved with ACEI/ARB use, which is an important predictor for AF, was unknown in these patients. Third, the baseline characteristics of the patients in the 3 groups were not completely matched, and selection bias might have confounded the result. Finally, this study mainly included East Asian subjects.

## CONCLUSIONS

Both ARB and ACEI, used either as a monotherapy or as a combined with other antihypertensive medication(s), can effectively reduce the risk of AF in hypertensive patients with or without risk factors for AF. ARB was more effective for AF prevention than ACEI, specifically in hypertensive patients with a history of prior stroke or TIA.
